# COVID-19 pandemic and physical health screening in an assertive community treatment service

**DOI:** 10.1192/j.eurpsy.2021.1767

**Published:** 2021-08-13

**Authors:** M. Taylor, I. Udo

**Affiliations:** 1 Schulich School Of Medicine & Dentistry, Western University, London, Canada; 2 Department Of Psychiatry, Western University, London, Canada

**Keywords:** Assertive community treatment, COVID-19, antipsychotic, Physical Health Screening

## Abstract

**Introduction:**

Patients with severe mental illness experience physical health inequities. They are less likely to receive preventative care and adequate treatment for physical illnesses. Physical health screening of patients receiving antipsychotics is usually carried out every six months. This comprises screening bloodwork and ECGs, and the sharing of results with family physicians.

**Objectives:**

We sought to investigate whether the pandemic affected the receipt of routine physical health screening in patients managed by an Assertive Community Treatment (ACT) Service.

**Methods:**

A comprehensive chart review was performed on 62 ACT patients. We compared the receipt of screening bloodwork and ECGs from March—December 2020 to the same period in 2019. Results were analyzed using McNemar’s Chi square test with Yates’ correction.

**Results:**

Patients were less likely to have received an ECG during the pandemic period. 69% received an ECG from March—December 2019 versus 42% from March—December 2020 (χ^2^= 7.76, p<0.01). Similarly, patients were less likely to have received screening bloodwork during the pandemic period (69% vs. 50%, Χ^2^= 4.32, p<0.05). Qualitative discussion with ACT staff regarding the 39 patients who had not received an ECG and/or bloodwork during the pandemic period revealed system-related (8%), patient-related (23%), and Covid-related (69%) barriers to screening. Covid-related barriers included transport concerns and lab closures.

**Conclusions:**

ACT patients were less likely to have received routine health screening during the Covid-19 pandemic. Thus, the pandemic may have exacerbated physical health inequities for patients with severe mental illness. Covid-related barriers to screening represent an important target for intervention.

**Disclosure:**

No significant relationships.

